# Human cystic echinococcosis detected in mesentery: A case report

**DOI:** 10.1177/2050313X241287645

**Published:** 2024-10-09

**Authors:** Hossein Schandiz, Salah NM Nasser, Bente Ekeberg, Mette K Pedersen, Truls M Leegaard, Torill Sauer

**Affiliations:** 1 Department of Pathology, Akershus University Hospital (Ahus), Akershus, Norway; 2 Faculty of Medicine, Institute of Clinical Medicine, University of Oslo, Campus Ahus, Oslo, Norway; 3 Department of Radiology, Akershus University Hospital (Ahus), Akershus, Norway; 4 Department of Microbiology and Infection Control, Akershus University Hospital (Ahus), Akershus, Norway

**Keywords:** Human cystic echinococcosis, hydatid disease, mesenteric cyst

## Abstract

Cystic echinococcosis, although rare in Europe, presents a diagnostic challenge when encountered, especially in atypical locations such as the mesentery. This case report is significant because it highlights the unique presentation of mesenteric hydatid cysts, emphasizing the importance of considering uncommon etiologies in differential diagnosis, particularly in immigrant populations. The novelty of this case lies in its rarity and the diagnostic dilemma it posed, ultimately leading to successful management through prompt recognition and accurate diagnosis. A 33-year-old pregnant female from East Africa presented with intermittent abdominal pain during pregnancy. Imaging revealed a cystic mass adjacent to the mesentery, initially misdiagnosed as an ovarian cyst. Postpartum, she developed acute abdominal symptoms, leading to a revised diagnosis of a ruptured hydatid cyst. Antiparasitic treatment and surgical intervention were initiated, resulting in successful management. This case underscores the necessity of prompt recognition and accurate diagnosis of rare conditions such as mesenteric hydatid cysts, particularly in immigrant populations. A multidisciplinary approach is crucial for optimal patient care in such cases.

## Introduction

Although relatively uncommon in many European countries, hydatid disease is more prevalent in the Mediterranean, Middle East, Central Asia, East Asia, East Africa, and South America.^1^ The Norwegian Institute of Public Health registers a few important cases (0–7) in patients every year; the majority are in the age range of 20–49 years.^2^ Human cystic echinococcosis (CE) is a parasitic disease caused by infection with *Echinococcus granulosus* senso lato. Approximately 95% of reported cases of human hydatid disease are caused by *E. granulosus*.^3^ Although hydatid cysts can be found in almost all tissues or organs in the human body, the liver (50%–77%), lungs (15%–47%), spleen (0.5%–8%), and kidneys (2%–4%) are the most commonly involved organs.^4^ We report a case of mesenteric hydatid cyst, originally misinterpreted as an ovarian cyst, in a preterm pregnant woman.

## Case presentation

**Figure 1.**
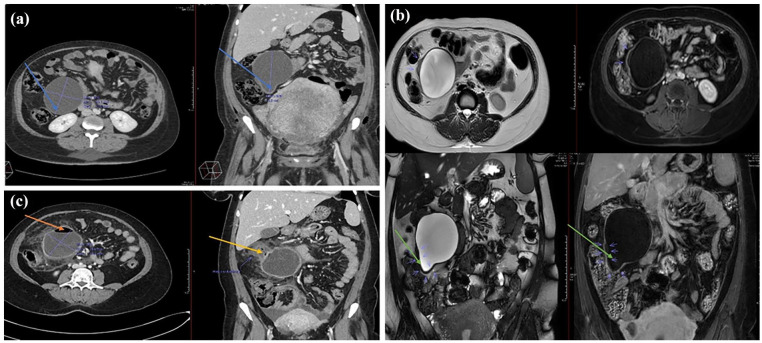
(a) CT scan showing the transverse section (left) and frontal view (right) of the abdomen. The cyst, with a caudal protrusion and focal wall thickening, was present on the right side of the abdomen (blue arrows). (b) MRI scan with transverse section (above) and frontal view (below) of the abdomen showing the cyst with a smooth wall and caudal protrusion with focal wall thickening (green arrows). The cyst contents were mostly homogenous with high T2 and low T1 signal intensities and were diagnosed as a benign cyst, possibly lymphangioma. (c) CT scan of the abdomen showing air content in the cyst (orange arrow on left image) and area of rupture and reactive inflammation (yellow arrow on right image).

**Figure 2.**
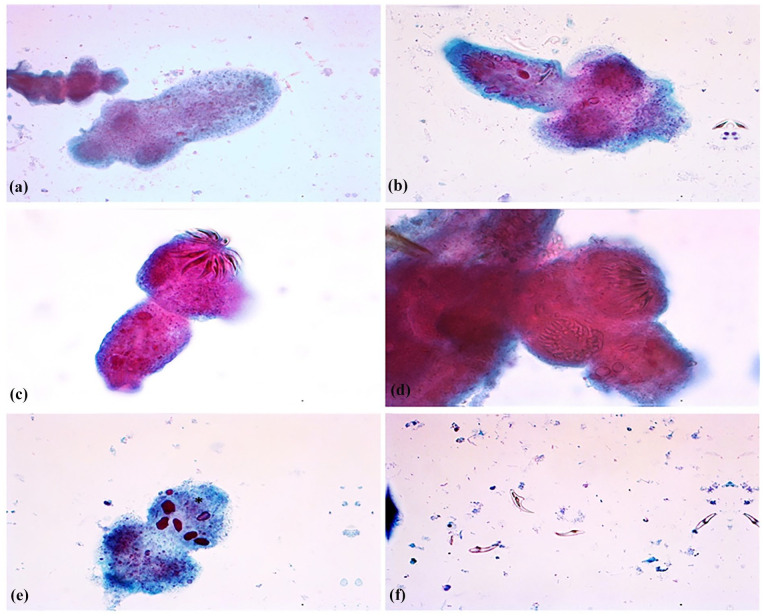
(a) Necrotic protoscoleces. LBC. PAP stain ×40. (b) Degenerated protoscoleces and free hooklets. LBC. PAP stain ×40. (c) and (d) Degenerated protoscoleces and preserved hooklets. LBC. PAP stain ×40. (e) Degenerated protoscoleces with eggs in immature proglottids with calcareous corpuscles (*). LBC. PAP stain ×40. (f) Free hooklets. LBC. PAP stain ×40. LBC: liquid-based cytology; PAP: Papanicolaou.

**Figure 3.**
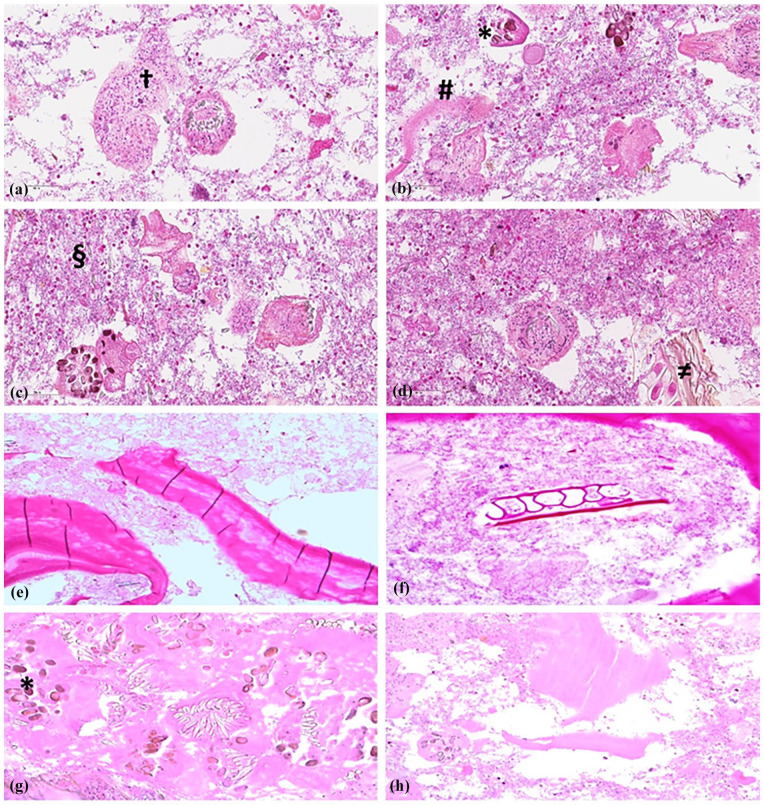
(a) Ample background debris with bacteria, necrotic protoscoleces, and degenerated protoscoleces with preserved hooklets (**†**). Cell block. HPS stain ×40. (b) Ample background debris with bacteria and free hooklets, necrotic and degenerated protoscoleces, immature proglottids with calcareous corpuscles (_*_) with eggs and laminated membranes (#). Cell block. HPS stain ×40. (c) Ample background debris with bacteria and free hooklets (§), necrotic and degenerated protoscoleces. Cell block. HPS stain ×40. (d) Ample background debris with bacteria, free hooklets, degenerated protoscoleces, and plant remnants (**≠**) were consistent with feces. Cell block. HPS stain ×40.(e) Overview of large-laminated membranes. Cell block. PAS stain ×20. (f) At the periphery and edge of a laminated membrane is seen. The background bacteria and remnant plant material were consistent with feces. Cell block. PAS stain ×20. (g) Multiple parts of the protoscoleces with hooklets and disrupted immature proglottids with calcareous corpuscles (*) with eggs. Cell block. PAS stain ×20. (h) Part of protoscoleces and laminated membrane material. In the background debris containing bacteria. PAS stain ×20. HPS: hematoxylin-phloxine-saffron; PAP: Papanicolaou.

The patient was a 33-year-old pregnant (gravida 4, para 1) immigrant female from East Africa. A routine ultrasound examination 2 months prior to the term showed an intra-abdominal cystic lesion measuring 8 cm × 8 cm in the right iliac fossa region. The patient was diagnosed as having an ovarian cyst that did not require immediate intervention. She had experienced intermittent abdominal pain and discomfort during the last week of pregnancy. She gave birth to a healthy child via a cesarean section. One week postpartum, owing to clinical suspicion of intestinal ileus, a CT scan (Figure 1(a)) was performed. The scan showed a large cystic mass that measured 10 cm × 9 cm × 8 cm in the right intra-abdominal region adjacent to the mesentery. The cyst leaned against the small and large intestines but did not originate from these organs. The cyst was not located near the right ovarium, appendix, kidney, or other intra-abdominal or pelvic organs. The origin of the cyst was uncertain, although it was assumed that the mesentery was the possible origin. A thick cyst wall and caudal contrast uptake were observed. The appearance was not compatible with abscess, hematoma, or seroma. An MRI (Figure 1(b)) that was conducted to elucidate the possible etiology and origin of the cyst displayed a large and unilocular cystic lesion on the right side of the abdominal cavity that measured 8.5 cm × 10.5 cm × 9.5 cm. The wall of the cyst was uniform with a thickness of 2–4 mm. In the caudolateral section of the cyst, a protrusion with an edematous wall 5 mm in thickness and contrast-enhanced uptake was observed. The contents of the cyst displayed high T2 and low T1 signal intensities, respectively, confirming a fluid-filled lesion. In the cyst wall, no nodular contrast uptake components were observed. There was no reduced infusion, but a focus on the lateral wall showed T2 translucence corresponding to the area with a high T2 signal. The cyst originated from the mesentery and not from the small or large intestine. The most common lesions in this anatomical location are lymphangiomas. Five weeks later, the patient was hospitalized because of an acute onset of abdominal pain, nausea, and vomiting. Clinical examination of the abdomen revealed distention and tenderness on palpation and pressure. Acute inflammatory biochemical markers in the blood, such as C-reactive protein and leukocytes, were elevated. A tentative diagnosis of peritonitis due to a ruptured cyst was made, and antibiotic treatment was initiated. A subsequent CT scan (Figure 1(c)) confirmed a ruptured cyst with wall thickening and inflammation of the surrounding fat tissue. The ascending colon displayed wall thickening and an ill-defined focus, suggestive of a rupture. The cyst was partially filled with air, consistent with an abscess. Intraperitoneal fluid was observed, particularly in the rectouterine pouch. A drain was installed, and two samples from the contaminated intraperitoneal fluid were submitted to the Department of Microbiology and Infection Control for microbiological analysis and to the Department of Pathology for cytological investigations, respectively. Primary microbiological evaluations at the former department were consistent with the presence of *Escherichia coli*. The fluid samples were used for cytological analysis at the Department of Pathology a few days later. The sample was centrifuged, the supernatant was discarded, and BD SurePath™ (SP) liquid-based cytology (LBC) reagent was added as a fixative and subjected to Papanicolaou (PAP) staining. Microscopic examination of the PAP-stained slides harvested from LBC material displayed abundant protoscoleces (Figure 2(a)–(f)) with both attached and scattered hooklets (Figure 2(a)–(e)) as well as numerous immature proglottids with calcareous corpuscles (Figure 2(e)). The remaining material was further processed in formalin-fixed paraffin-embedded cell blocks and stained with hematoxylin-phloxine-saffron (HPS) and periodic acid-schiff (PAS). Examinations of the HPS-stained slides revealed also abundant protoscoleces (Figure 3(a)–(e)) with both attached and scattered hooklets. Both HPS and PAS-stained slides showed abundant eosinophilic sheets of laminated membranes (Figure 3(b), (e), (f), and (g)) and numerous immature proglottids (Figure 3(b) and (h)).

These findings were consistent with hydatid cysts (*Echinococcus*) and confirmed the preliminary diagnosis. Following this, the previous microbiological diagnosis was re-evaluated, and hooklets were identified by microscopic analysis. In the background, degenerative and necrotic tissues, along with rod-shaped bacteria and remnants of plant material, as found in the feces, were observed (Figure 3(d)). Based on these findings, the on-call clinician was immediately notified and antiparasitic treatment (albendazole) was initiated at the Department of Infectious Diseases.

The patient underwent a right hemicolectomy within a few days at another hospital. The cyst, located in the mesentery, was surrounded by massive inflammation and was close to the right colic flexure. The surgeon did not detect any further intra-abdominal cysts. Gross examination of surgical specimens disclosed a cystic structure in the mesentery. Histopathological assessment of the cyst revealed fat tissue necrosis, inflammation consisting of granulocytes and macrophages, laminated acellular formations, and remnants of the plant material. A focus on abundant fibrosis and chronic inflammation was also present. Eosinophilic lamellae and compact shapes were observed in the necrotic areas. There was no evidence of vital protoscolex on the examined slides. Fibrous obliteration was found in the distal section of the appendix. Other areas of the colon and distal ileum were unremarkable.

To verify the diagnosis, the samples were sent to the Norwegian Reference Laboratory for antibody analysis using an enzyme-linked immunosorbent assay (ELISA) and Western blot (WB). ELISA showed a borderline immunoglobulin G (IgG) reaction (0.9); however, IgG antibodies were detected by WB. Finally, *E. granulosus* DNA was identified using PCR, which confirmed the previous diagnosis. Clinical follow-up of the patient was unremarkable in the following weeks and months.

## Discussion

Hydatid cysts are known to occur in the mesentery^5–8^ although this location is uncommon. Primary peritoneal hydatid cysts account for 2% of all intra-abdominal hydatidosis cases.^9^ Balik et al.^6^ found that only 1 out of 27 intra-abdominal and extrahepatic hydatid cysts were located in the mesentery. Intraperitoneal hydatid cysts are usually secondary to the rupture of a primary hepatic or splenic cyst.^9^ In the mesentery, they may present as nonspecific masses with accompanying pain due to traction or pressure on surrounding structures. Ultrasound is usually the first-line imaging modality, followed by CT scan, to obtain more precise information about the size, location, adjacent organs, and number of cysts. Based on radiological findings, CE can be divided into four subtypes: simple cysts without detectable intracystic content, daughter cysts, calcified cysts, and complex cysts (infected or ruptured), respectively. The World Health Organization has classified CE into three relevant groups: active (CE1 and 2), transitional (CE3), and inactive (CE4 and 5).^10,11^ On the initial CT scan, a unilocular cyst with irregular cyst wall thickness was observed. Thickening of the wall of the cyst is uncommon in mesenteric cysts. However, the radiological findings were not specific to CE, and clinical information was essential to raise awareness of this diagnosis. Cytological findings with abundant protoscoleces and hooklets were specific and pathognomonic for CE.^12,13^ Eventually, *E. granulosus* DNA was detected and identified using PCR, which confirmed the diagnosis.^14^ The findings of remnants of the plant material in cytological and histopathological examinations along with the detection of *Escherichia coli* in microbiological analysis were consistent with contamination from intestinal content, caused by perforation, although visible defects in large intestine wall were not notified in gross examination of the surgical specimen. We had no information regarding when this rupture occurred. Parasitic diseases are uncommon in Northern European countries and are usually imported from other parts of the world. Nevertheless, in recent years, a large immigration from the Middle East (e.g., Syria) to many European countries, including Norway, may contribute to a higher incidence of this disease. They are rarely observed in cytological diagnostic samples, resulting in a limited diagnostic experience. If a hydatid cyst based on clinical investigations is suspected, the general rule is to avoid puncturing the cyst to prevent possible anaphylactic reactions. In this patient, the cyst ruptured spontaneously. Hydatid cysts grow slowly with an annual growth rate of 1–10 mm^5^ and can be asymptomatic, until reaching an extent that triggers clinical signs.^1,2^ The incubation period varies from one to several years.^1,2^ humans are infected through direct or indirect contact with the definitive host of the parasite, that is, dogs. This patient had not visited her home country in the last 5 years and had no information about when she might have been infected.

## Conclusions

This case report underscores the diagnostic challenge posed by mesenteric hydatid cysts, especially when presenting as atypical intra-abdominal masses. The successful management of this case highlights the critical importance of prompt recognition and accurate diagnosis, particularly in immigrant populations from endemic regions. Additionally, it emphasizes the necessity of a multidisciplinary approach involving imaging specialists, pathologists, and infectious disease experts to ensure optimal patient care. Importantly, this case serves as a reminder for clinicians to maintain a high index of suspicion for rare parasitic diseases, even in non-endemic areas. Further dissemination of such cases through top medical journals can enhance awareness and contribute to improved clinical outcomes in similar scenarios.
